# Supplementary material for: Untargeted Microbial Exometabolomics and Metabolomics Analysis of Helicobacter pylori J99 and jhp0106 Mutant
Source: Metabolites. 2021 Nov 28;11(12):808. doi: 10.3390/metabo11120808 (PMC8707867; doi:10.3390/metabo11120808)
Supplement: Supplementary file 1 [file metabolites-11-00808-s001.zip › metabolites-1462504-supplementary.pdf]

## Supporting Information

*Article*

# Untargeted Microbial Exometabolomics and Metabolomics Analysis of Helico-bacter pylori J99 and jhp0106 Mutant

**Cheng-Yen Kao [1], Pei-Yun Kuo [1] and Hsiao-Wei Liao [2,*]**

## Table of Contents

Identification of the tentatively identified compounds.................................... S-2
Figure S1........................................................................................ S-3
Figure S2........................................................................................ S-4
Figure S3........................................................................................ S-5
Figure S4........................................................................................ S-6
Figure S5........................................................................................ S-7
Figure S6........................................................................................ S-8
Figure S7........................................................................................ S-9
Figure S8........................................................................................ S-10
Table S1 ........................................................................................ S-11

**Identification of the tentatively identified compounds**

The use of reference standards is helpful for chemical identification, however, some of the identified compounds are not commercially available. Among these tentatively identified compounds, phosphorylethanolamine, PE(37:1), and TG(49:8) have lower abundance which hindered the obtainment of the MS2 spectrum. For this reason, according to the identified features we purchased three reference standards (cPA 16:0, DG 16:0/16:0, and PE 16:0/16:0) to confirm the tentatively identified compounds by using the retention time, accurate *m/z*, and MS2 spectrum from these reference standards.

The reference standards showed the same retention time and accurate *m/z* as the tentatively identified compounds of cPA 16:0, DG 32:0, and PE 32:0. The MS2 spectrum of the reference standards (cPA 16:0, DG 16:0/16:0, and PE 16:0/16:0) and the tentatively identified compounds were provided in Fig S3 to Fig S8. Among these tentatively identified compounds, cPAs are cyclic LPAs. For identification of LPAs and cPAs, the fragment of m/z 153 is a common fragment spectrum from LPAs and CPAs, and sometimes the fragment of fatty acids could be also observed in the MS2 of LPAs and CPAs in the negative mode. For identification of PEs, the fragment of neutral loss of *m/z* 141 for PEs could be observed in the positive mode. For the identification of DGs, the fragments of fatty acid side chains could be observed in the MS2 spectrum.

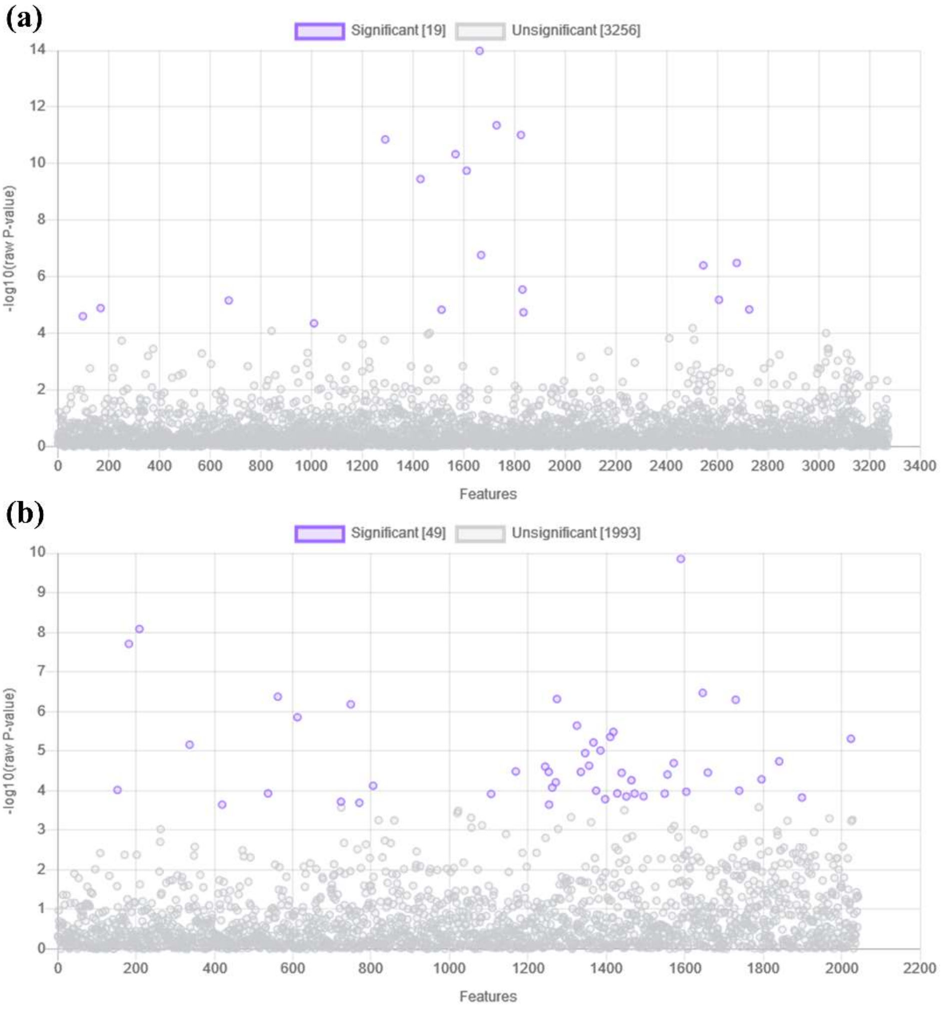


Figure S1. Two-sample t-tests of features in culture media (a) and *H. pylori* pellet (b) with a FDR corrected *p* value cut-off of 0.01.

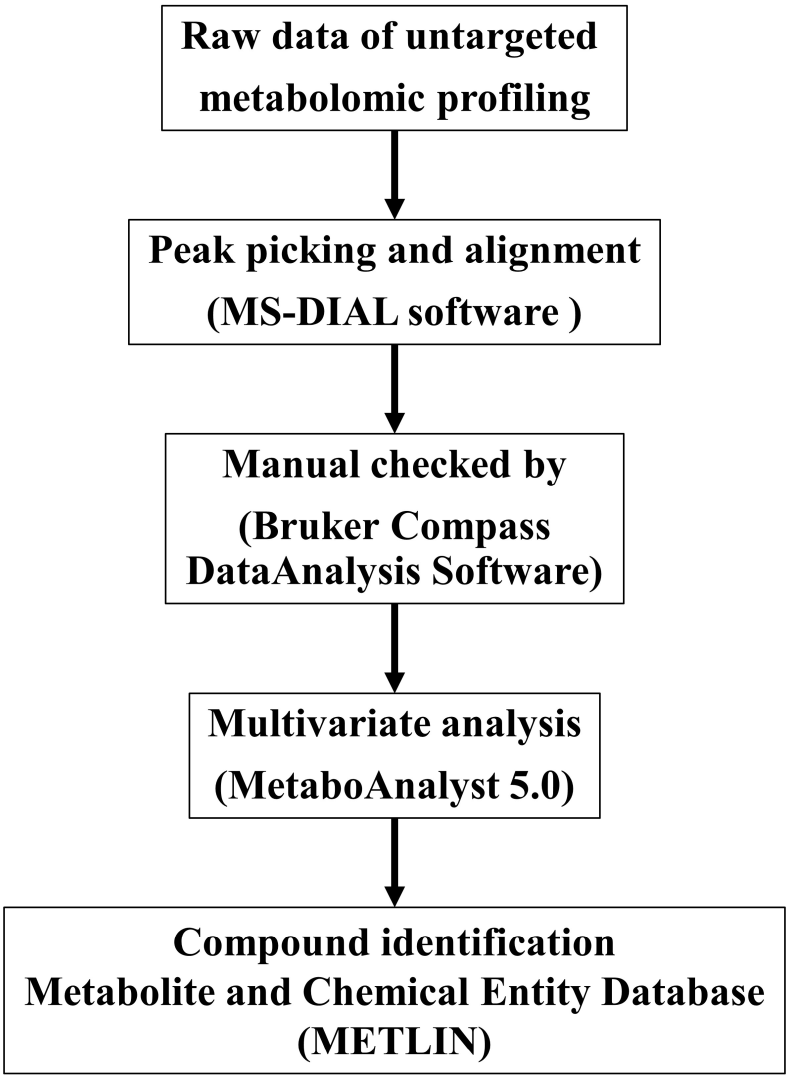


Figure S2. The flow chart of data analysis workflow in this study.

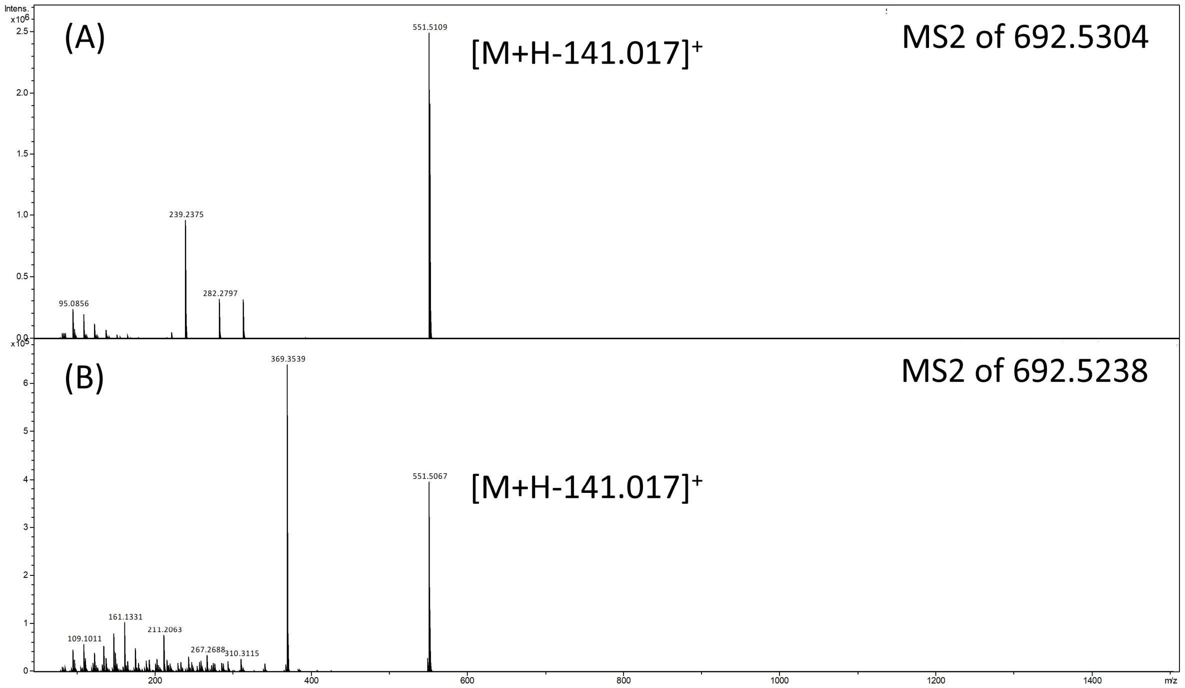


Figure S3. The obtained MS2 fragment of (A) reference standard of PE (16:0/16:0) and (B) PE 32:0 from the culture media of *H. pylori.*

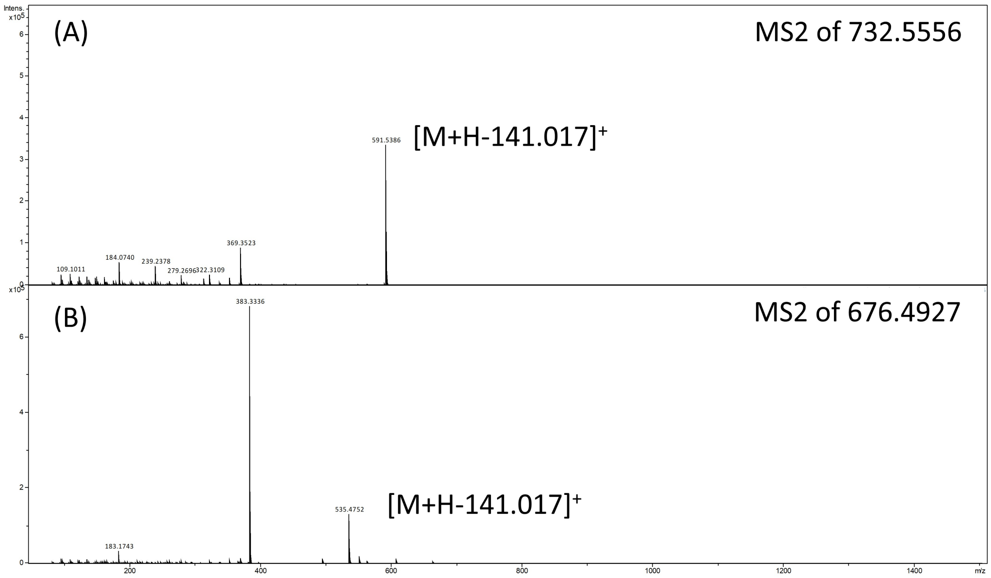


Figure S4. The obtained MS2 fragment of (A) PE 35:1 and (B) PE 31:1 from the culture media of *H. pylori.*

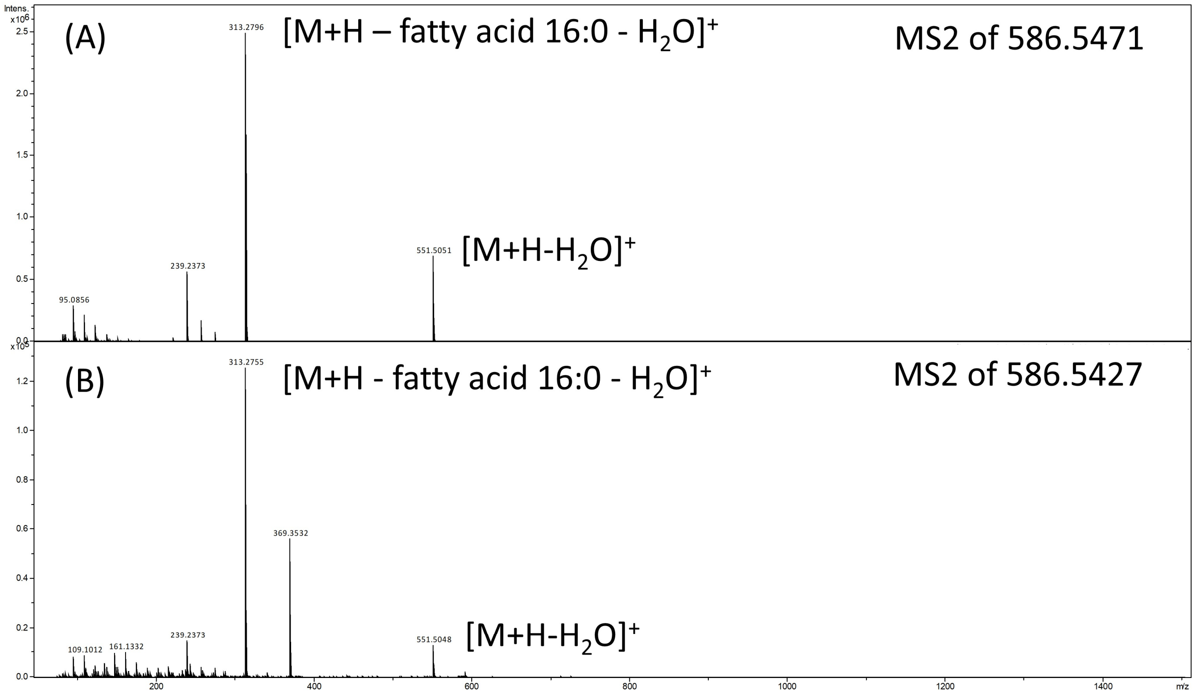


Figure S5. The obtained MS2 fragment of (A) reference standard of DG (16:0/16:0) and (B) DG 32:0 from *H. pylori* pellet.

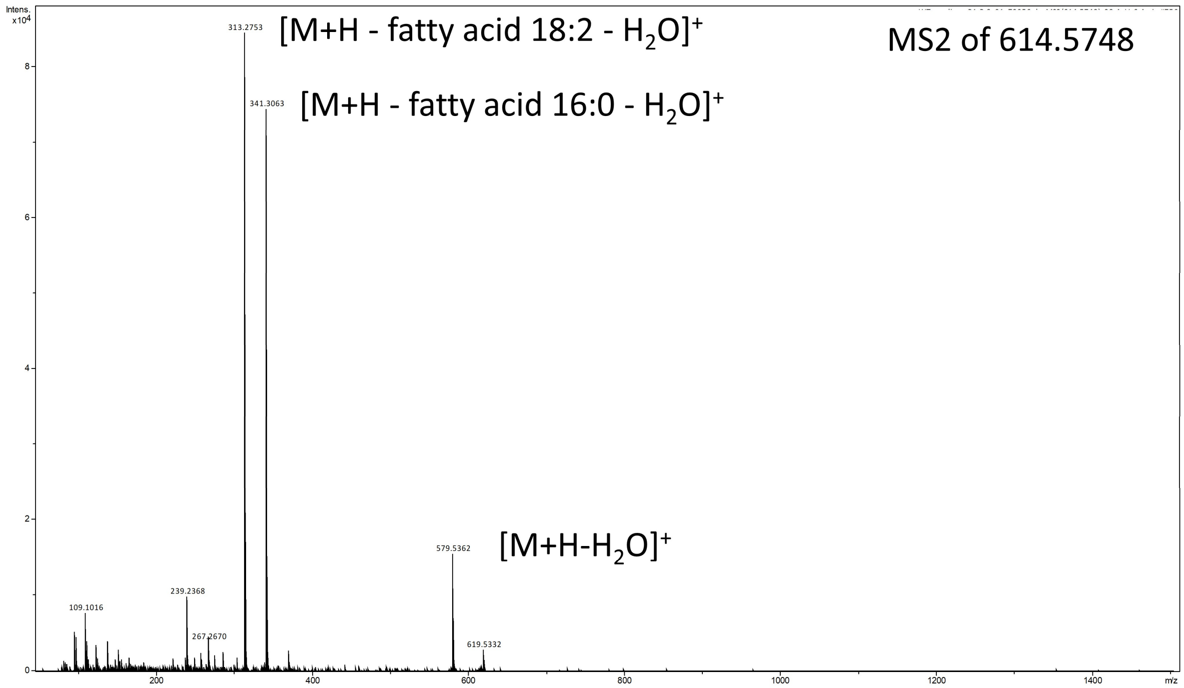


Figure S6. The obtained MS2 fragment of DG 34:0 from *H. pylori* pellet.

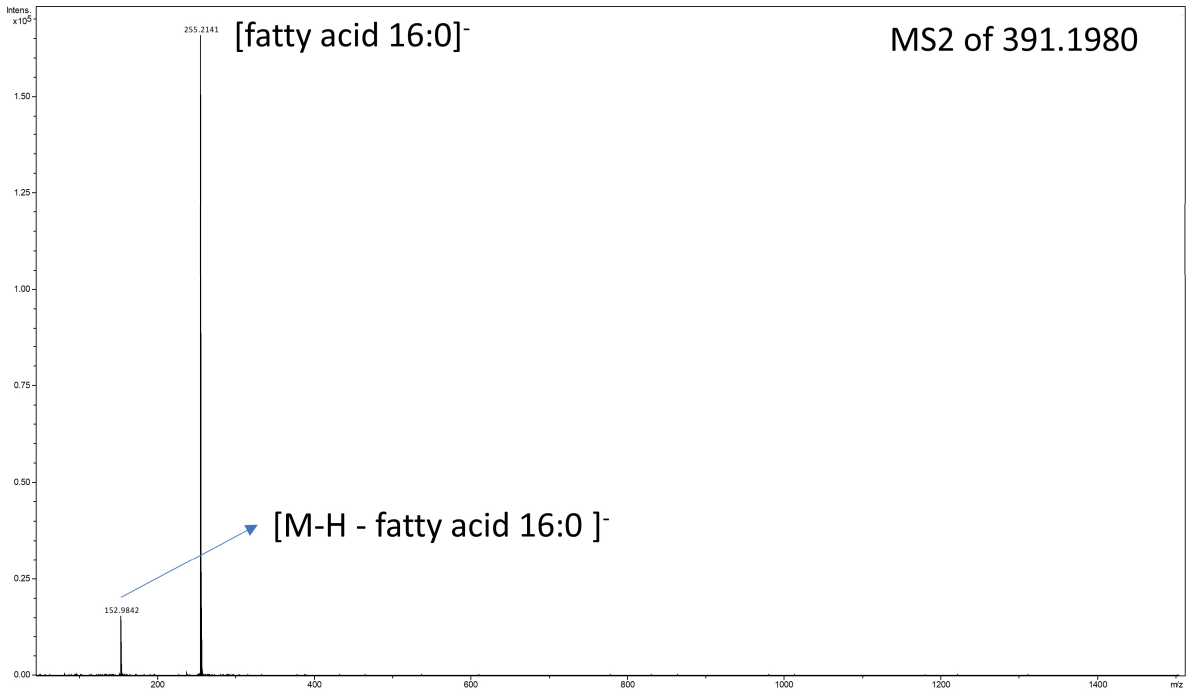


Figure S7. The obtained MS2 fragment of cPA 16:0 reference standard.

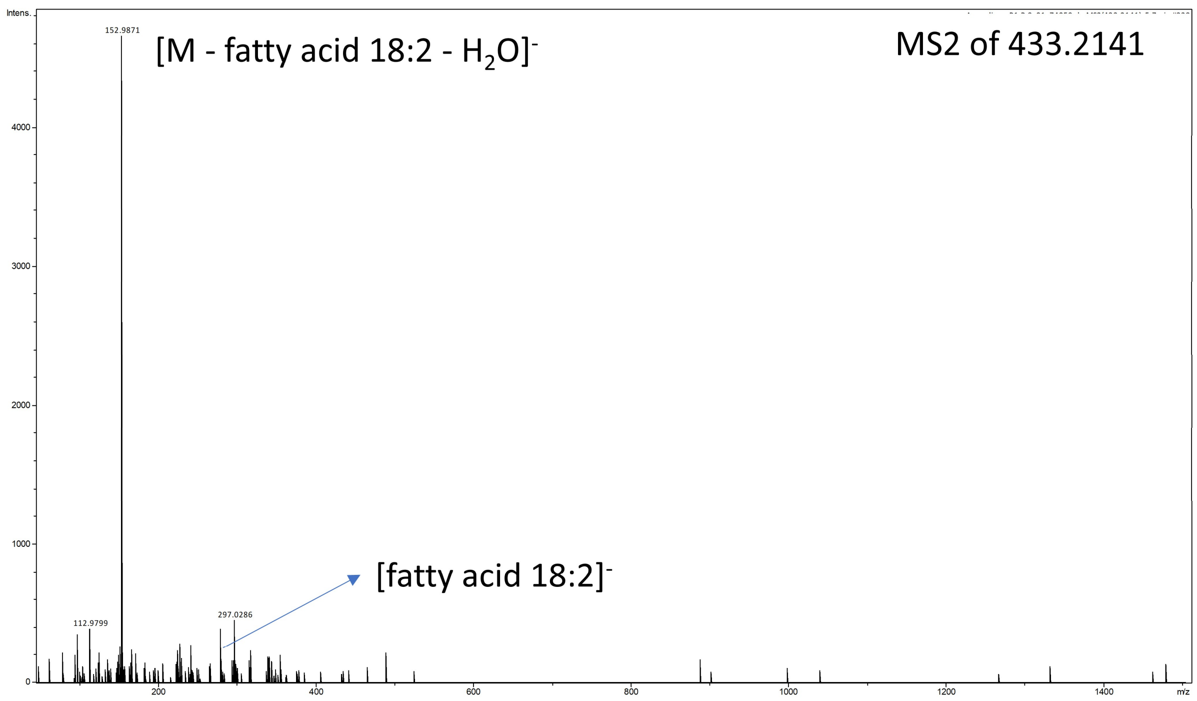


Figure S8. The obtained MS2 fragment of LPA 18:2 from the culture media of *H. pylori*.

Table S1. The detailed information of metabolites for extraction efficiency test, and tentatively identified from the culture media and *H. pylori* pellet.

| Detected m/z | Theoretical m/z | ppm error | Retention time (min) | Formula | Adduct | Annotation |
|---|---|---|---|---|---|---|
| 90.0546 | 90.0550 | -4.3 | 0.7 | C3H7NO2 | M+H | Alanine |
| 104.1070 | 104.107 | -0.3 | 0.6 | C5H13NO | M+H | Choline |
| 114.0663 | 114.0662 | 0.4 | 0.7 | C4H7N3O | M+H | Creatinine |
| 116.0705 | 116.0706 | -1.2 | 0.7 | C5H9NO2 | M+H | Proline |
| 118.0860 | 118.0863 | -2.7 | 0.7 | C5H11NO2 | M+H | Valine |
| 121.0886 | 121.0876 | 8.3 | 0.7 | $^{13}$C5H9NO2 | M+H | Proline-$^{13}$C5 (SIL-IS) |
| 123.1036 | 123.1033 | 2.5 | 0.7 | $^{13}$C5H11NO2 | M+H | Valine-$^{13}$C5 (SIL-IS) |
| 142.0271 | 142.0264 | 4.7 | 6.4 | C2H8NO4P | M+H | Phosphorylethanolamine |
| 166.0868 | 166.0868 | -0.2 | 1.7 | C9H11NO2 | M+H | Phenylalanine |
| 172.1076 | 172.1072 | 2.1 | 1.7 | $^{13}$C6C3H11NO2 | M+H | Phenylalanine-$^{13}$C6 (SIL-IS) |
| 175.1186 | 175.1190 | -2.3 | 0.6 | C6H14N4O2 | M+H | Arginine |
| 181.1402 | 181.1394 | 4.3 | 0.6 | $^{13}$C6H14N4O2 | M+H | Arginine-$^{13}$C6 (SIL-IS) |
| 182.0805 | 182.0817 | -6.8 | 1.2 | C9H11NO3 | M+H | Tyrosine |
| 188.1015 | 188.1021 | -3.2 | 1.3 | $^{13}$C6C3H11NO3 | M+H | Tyrosine-$^{13}$C6 (SIL-IS) |
| 205.0963 | 205.0977 | -7.0 | 4.1 | C11H12N2O2 | M+H | Tryptophan |
| 365.2096 | 365.2111 | -4.0 | 5.6 | C24H28O3 | M+H | Unknown |
| 393.2403 | 393.2401 | 0.4 | 5.7 | C19H37O6P | M+H | CPA 16:0 |
| 404.3892 | 404.3887 | 1.3 | 6.7 | C27H46O | $M+NH_4$ | Cholesterol |
| 405.1987 | 405.1980 | 1.8 | 5.6 | C16H28N4O8 | M+H | Unknown |
| 417.2403 | 417.2406 | -0.6 | 5.7 | C21H39O7P | $M+H-H_2O$ | LPA 18:2 |
| 435.2518 | 435.2506 | 2.8 | 5.6 | C21H39O7P | M+H | LPA 18:2 |
| 457.2323 | 457.2331 | -1.7 | 5.7 | C21H39O7P | $M+NH_4$ | LPA 18:2 |
| 478.2942 | 478.2928 | 3.0 | 5.7 | C23H44NO7P | M+H | LysoPE 18:2 |
| 496.3393 | 496.3398 | -1.0 | 6.7 | C24H50NO7P | M+H | LysoPC 16:0 |
| 518.3260 | 518.3241 | 3.6 | 5.8 | C26H48NO7P | M+H | LysoPC 18:3 |
| 522.3566 | 522.3554 | 2.2 | 6.3 | C26H52NO7P | M+H | LysoPC 18:1 (IS) |
| 524.3702 | 524.3711 | -1.6 | 7.0 | C26H54NO7P | M+H | LysoPC 18:0 |
| 551.5086 | 551.5039 | 8.5 | 6.3 | C35H68O5 | $M+H-H_2O$ | DG 32:0 |
| 569.5173 | 569.5140 | 5.8 | 6.3 | C35H68O5 | M+H | DG 32:0 |
| 579.5409 | 579.5352 | 9.8 | 6.4 | C37H72O5 | $M+H-H_2O$ | DG 34:0 |
| 586.5443 | 586.5405 | 6.5 | 6.3 | C35H68O5 | $M+NH_4$ | DG 32:0 |
| 597.5514 | 597.5453 | 10.2 | 6.4 | C37H72O5 | M+H | DG 34:0 |
| 614.5759 | 614.5718 | 6.7 | 6.4 | C37H72O5 | $M+NH_4$ | DG 34:0 |
| 636.4614 | 636.4598 | 2.6 | 6.1 | C33H66NO8P | M+H | PE 28:0 |

| | | | | | | |
|---|---|---|---|---|---|---|
| 676.4938 | 676.4912 | 3.8 | 6.1 | C36H70NO8P | M+H | PE 31:1 |
| 692.5238 | 692.5224 | 2.0 | 6.3 | C37H74NO8P | M+H | PE 32:0 |
| 704.5197 | 704.5225 | -4.0 | 6.2 | C38H74NO8P | M+H | PE 15:0/18:1 (IS) |
| 716.6771 | 716.6704 | 9.4 | 6.4 | C50H82O | $M+NH_4$ | Unknown |
| 732.5565 | 732.5538 | 3.7 | 6.3 | C40H78NO8P | M+H | PE 35:1 |
| 760.5859 | 760.5856 | 0.4 | 6.4 | C42H82NO8P | M+H | PE 37:1 |
| 760.5855 | 760.5851 | 0.5 | 10.1 | C42H82NO8P | M+H | PC 34:1 |
| 780.5510 | 780.5538 | -3.6 | 7.9 | C44H78NO8P | M+H | PC 36:5 |
| 782.5704 | 782.5694 | 1.3 | 8.8 | C44H80NO8P | M+H | PC 36:4 |
| 784.5861 | 784.5851 | 1.2 | 9.3 | C44H82NO8P | M+H | PC 36:3 |
| 804.5473 | 804.5538 | -8.1 | 7.7 | C46H78NO8P | M+H | PC 38:7 |
| 822.6606 | 822.6606 | 0.0 | 6.4 | C52H84O6 | $M+NH_4$ | TG 49:8 |
| 868.7419 | 868.7389 | 3.5 | 6.8 | C55H94O6 | $M+NH_4$ | TG 52:6 |
| 880.5858 | 880.5910 | -6.0 | 6.0 | C45H83O13P | $M+NH_4$ | PI 36:2 |
| 890.7251 | 890.7232 | 2.1 | 6.6 | C57H92O6 | $M+NH_4$ | TG 54:9 |
| 892.7383 | 892.7388 | -0.6 | 6.7 | C57H94O6 | $M+NH_4$ | TG 54:8 |

* All putative identified names were level 3 identification (tentatively characterized compound classes) by matching precursor *m/z* to Metlin database.

Table S2. The selected SIL-IS and IS for matrix effect correction in the figure 1.

| **Tentatively identified metabolites** | **IS** |
|---|---|
| Choline | Proline-$^{13}C5$ (SIL-IS) |
| Creatinine | Proline-$^{13}C5$ (SIL-IS) |
| Proline | Proline-$^{13}C5$ (SIL-IS) |
| Phenylalanine | Phenylalanine-$^{13}C6$ (SIL-IS) |
| Arginine | Arginine-$^{13}C6$ (SIL-IS) |
| Tyrosine | Tyrosine-$^{13}C6$ (SIL-IS) |
| Tryptophan | PE 15:0/18:1 (IS) |
| Cholesterol | PE 15:0/18:1 (IS) |
| LysoPE 18:2 | PE 15:0/18:1 (IS) |
| LysoPC 16:0 | LysoPC 18:1 (IS) |
| LysoPC 18:3 | LysoPC 18:1 (IS) |
| LysoPC 18:0 | LysoPC 18:1 (IS) |
| PE 28:0 | PE 15:0/18:1 (IS) |
| PE 35:1 | PE 15:0/18:1 (IS) |
| PE 38:6 | PE 15:0/18:1 (IS) |
| PC 34:1 | LysoPC 18:1 (IS) |
| PC 36:5 | LysoPC 18:1 (IS) |
| PC 36:4 | LysoPC 18:1 (IS) |
| PC 36:3 | LysoPC 18:1 (IS) |
| PC 38:7 | LysoPC 18:1 (IS) |
| TG 52:6 | LysoPC 18:1 (IS) |
| PI 36:2 | LysoPC 18:1 (IS) |
| TG 54:9 | LysoPC 18:1 (IS) |
| TG 54:8 | LysoPC 18:1 (IS) |
